# Role of APP in neuronal function

**DOI:** 10.1186/1750-1326-8-S1-O4

**Published:** 2013-09-13

**Authors:** Hui Zheng

**Affiliations:** 1 Huffington Center on Aging, Department of Molecular and Human Genetics, Baylor College of Medicine, Houston, Texas, USA

## Background

Genetic and biochemical studies establish a central role of the amyloid precursor protein (APP) in Alzheimer’s disease (AD): Genetic mutations and gene amplification of *APP* are linked to early onset of familial Alzheimer’s disease (FAD); APP processing generates β-amyloid (Aβ) peptides, which are the principal components of the amyloid pathology. Therefore, understanding the role of APP in neuronal function and dysfunction will provide crucial insights to AD pathogenesis.

## Results

We have a long-standing interest in studying the physiological functions of APP in neurons and synapses. Analysis of various loss-of-function mutants of APP and combining with a mixed-culture system allowed us to identify APP as a synaptic adhesion protein. We recently uncovered a potent role of APP in mediating adult neurogenesis in dentate gyrus: Loss of APP results in an aberrant increase in progenitor proliferation but impaired newborn neuron differentation, maturation and integration. Intriguingly, we found that APP is highly expressed in GABAergic interneurons and that specific deletion of APP in these neurons, but not in excitatory neurons, leads to similar neurogenesis defect. Our mechanistic and functional studies indicate that this activity is mediated by a general role of APP in regulating GABA release through its synaptic adhesion property.

## Conclusion

AD is an age-related disease. Adult neurogenesis declines sharply with aging. Increasing evidence supports a role of hippocampal adult neurogenesis in brain function and its impairment in AD. Therefore, perturbation of APP-mediated adult neurogenesis may contribute to neuronal dysfunction and AD pathogenesis.

